# Impact of combined intermittent preventive treatment of malaria and helminths on anaemia, sustained attention, and recall in Northern Ghanaian schoolchildren

**DOI:** 10.3402/gha.v9.32197

**Published:** 2016-09-14

**Authors:** Ernest Cudjoe Opoku, Annette Olsen, Edmund Browne, Abraham Hodgson, John K. Awoonor-Williams, Lawrence Yelifari, John Williams, Pascal Magnussen

**Affiliations:** 1 Navrongo Health Research Centre, Ghana Health Service, Navrongo, Ghana; 2 Copenhagen School of Global Health, Faculty of Health and Medical Sciences, University of Copenhagen, Copenhagen, Denmark; 3 Department of Veterinary Disease Biology, Faculty of Health and Medical Sciences, University of Copenhagen, Frederiksberg, Denmark; 4 Department of Community Health, School of Medical Sciences, Kwame Nkrumah University of Science and Technology, Kumasi, Ghana; 5 Regional Health Directorate, Ghana Health Service, Bolgatanga, Ghana; 6 Centre for Medical Parasitology, Faculty of Health and Medical Sciences, University of Copenhagen, Copenhagen, Denmark

**Keywords:** co-administered intermittent preventive treatment of malaria and deworming, anaemia, sustained attention, recall, schoolchildren

## Abstract

**Background:**

The benefits of integrated control of malaria, schistosomiasis, and soil-transmitted helminth infections have not been fully explored in Ghanaian schoolchildren.

**Objective:**

To assess the impact of co-administered artemether-lumefantrine plus albendazole, and artemether-lumefantrine plus albendazole plus praziquantel compared to albendazole plus praziquantel on anaemia, sustained attention, and recall in schoolchildren.

**Design:**

This three-arm, open-label intervention study was carried out in Ghana among class three schoolchildren. Artemether-lumefantrine and albendazole were co-administered to 131 schoolchildren in Study Arm 1; artemether-lumefantrine, albendazole, and praziquantel to 90 children in Study Arm 2 versus albendazole and praziquantel to 127 children in Control Arm 3. Medicines were administered to all children at least 30 min after a meal. A HemoCue^®^ photometer was used to measure haemoglobin (Hb), while the code transmission test (CTT), adapted from the Test of Everyday Attention for Children (TEA-Ch), was used to measure sustained attention and recall before-and-after interventions in June 2011 and June 2012.

**Results:**

We observed significant malaria parasite prevalence reductions of 62.8 and 59.2% in Study Arm 1 from 24.2 to 9.0%, *p*<0.01, and 59.2% in Study Arm 2 from 26.7 to 10.9%, *p*<0.01), respectively, compared to 8.93% in Control Arm 3 (from 34.7 to 31.6%, *p*>0.05). Meanwhile, anaemia prevalence reduced significantly (*p*<0.01) in all three study arms after interventions by 38.4% (from 19.8 to 12.2%), 20.7% (from 26.6 to 21.1%), and 36.0% (from 28.3 to 18.1%) in Study Arms 1, 2, and 3, respectively. Although the interventions had no significant effects on Hb levels, anaemia prevalence reduced insignificantly by 38.4 and 20.7% in Study Arms 1 and 2, respectively, compared to 36.0% in Control Arm 3. Among schoolchildren in Study Arms 1 and 2, mean CTT score improved significantly after interventions by 10.4% (from 3.18 to 3.55, *p*=0.01) and 20.5% (from 2.83 to 3.56, *p*=0.01) respectively, compared to 5.75% in Control Arm 3 (from 2.95 to 3.13, *p*=0.09). Likewise, mean recall test score improvements after interventions were 16.9% (from 2.07 to 2.49, *p*=0.01) and 27.9% (from 1.91 to 2.65, *p*=0.01) in Study Arms 1 and 2, respectively, compared to 18.3% (from 1.92 to 2.35, *p*=0.01) in Control Arm 3.

**Conclusion:**

Combined intermittent preventive treatment of malaria and deworming reduced prevalence of anaemia and improved sustained attention and recall in schoolchildren. Best results for sustained attention and recall were seen in Study Arm 2.

## Introduction

Malaria is a major public health challenge in sub-Saharan Africa, is associated with high morbidity and mortality, and affects economic growth and development (1, 2). However, school-age children, who are most susceptible to schistosomiasis and soil-transmitted helminth (STH) infections, tend to harbour malaria parasites without symptoms in high malaria transmission regions (3–5). Repeated attacks of uncomplicated malaria and chronic asymptomatic malaria parasitaemia in school-age children are common and may result in anaemia, stunting, school absenteeism, and reduced cognitive abilities in schoolchildren in high transmission regions (5, 6). Yet, many malaria control programmes in sub-Saharan Africa lack policy and guidelines on how asymptomatic malaria should be dealt with in the most at-risk group: schoolchildren. Although the targeted population in the World Health Organization's (WHO) strategies and guidelines for the control of schistosomiasis and STHs through chemotherapy is school-age children (3, 4, 7), the global malaria chemoprevention strategy targets pregnant women and children under the age of 5 years (1). Tackling asymptomatic malaria parasitaemia in school-age children is not only right, appropriate, and ethical, but also relevant in the light of global plans to attain malaria elimination and eradication (2, 5, 6, 8).

Intermittent preventive treatment (IPT) of malaria was proposed as one of the strategies schools and health managers in high malaria transmission regions could use to deal with uncomplicated and asymptomatic malaria in schoolchildren (2, 5, 6, 9). IPT is the term used to describe the administration of a full curative dose of an antimalarial to a selected, target population at specified times without determining whether or not the subjects are infected (9). Previous studies have shown that an IPT programme in schools benefits schoolchildren's health and learning capability (9–12), and artemisinin-based combination therapy (ACT) has been used successfully for IPT in malaria endemic countries such as Mali, Uganda, and Ghana (13–15). Moreover, previous studies have shown that when an ACT is used in an IPT programme in an area where malaria and schistosomiasis coexist, the added benefit of schistosomiasis control could be derived (16, 17). However, these findings have not been fully translated into policy to benefit children across sub-Saharan Africa, even though there could be positive results integrating the control of neglected parasitic diseases and malaria (18–21).

In Ghana, most disease control activities implemented by the Ghana Health Service (GHS), including neglected tropical diseases (NTDs), malaria, HIV/AIDS, and tuberculosis, are donor-funded and run as vertical programmes. Even though they are associated with successes, most vertical control programmes are known to incorporate donor interests and dictates and political economics. Apart from addressing some aspects of the specific disease entity, they rarely lead to the strengthening of health systems and have been associated with health system fragmentation and disruption of health services (18, 22–24).

Following attainment of lower middle income status, Ghana has been experiencing dwindling donor support for its health sector, and funding support from the government of Ghana to the GHS has been reduced significantly (24). Recently, the GHS and its health partners have advocated for integrated programming. One successful integrated programme has been NTDs control, with regular distribution of ivermectin, praziquantel, and albendazole/mebendazole against lymphatic filariasis, onchocerciasis, schistosomiasis, and STH based on WHO guidelines (7). However, the control of malaria has not been integrated with the control of the aforementioned NTDs. Little or no knowledge exists regarding how integrated control of malaria, schistosomiasis, and STHs could benefit the most at-risk populations – school-age children, and there is a lack of policy and guidelines regarding how this integrated approach could work in Ghana. Having a policy framework to guide the integrated control of parasitic infections through the use of anti-parasitic drugs is vital. Some of the key ingredients required are 1) an all-encompassing policy to guide implementation, 2) evidence that integrated control benefits the targeted population, 3) evidence of safety and tolerability of the co-administered anti-parasitic drugs, and 4) cost effectiveness (3, 4, 18–21).

With the objective of providing evidence to support the development of an integrated policy for the control of malaria, schistosomiasis, and STHs in schoolchildren using chemotherapy, this study, as a part of a larger investigation, sought to determine whether IPT targeted at the high malaria transmission season (rainy season), combined with deworming, would reduce the prevalence of malaria parasitaemia and anaemia as well as improve sustained attention and recall in schoolchildren. This study therefore attempted to answer the research question: to what extent would co-administered IPT with artemether-lumefantrine and albendazole plus or minus praziquantel reduce anaemia prevalence and improve sustained attention and recall in schoolchildren when compared with albendazole plus praziquantel?

## Methods

### Study area and population

The study was carried out in six primary schools in the Kassena-Nankana Districts (KNDs) of the Upper East Region, Ghana, where poverty, malnutrition, anaemia, and malaria represent major public health challenges (25, 26). The KNDs lie within the Guinea Savannah woodlands between latitudes 10°30’–11°00’ N and longitudes 1°00’–1°30’ W. The KNDs have a population of about 150,000 inhabitants dispersed in mostly rural settings. The main occupation of the people is subsistence farming (millet, rice, sorghum, and livestock) and petty trading. On average, annual rainfall is about 850–950 mm (almost all in the months of May to September). The average annual temperature ranges from 18 to 45°C. Mosquito-breeding sites are found all year round due to the presence of a major irrigation dam (Tono Irrigation Project) and numerous smaller dams throughout the KNDs.

Malaria is caused predominantly by *Plasmodium falciparum* and is transmitted by *Anopheles gambiae, An. Funestus*, and *An. arabiensis* mosquito vectors in Ghana. Malaria transmission is most intense in the KNDs during the rainy season (May to November), but the entomological inoculation rate varies across Ghana's southern, forest, and northern savannah ecological zones (27–29). Previous studies in the KNDs have described a high incidence of malaria and anaemia in children and large numbers of school days lost due to malarial infection (30–32).

Ghana's National Malaria Control Programme (NMCP) implements a strategic plan and, over the past 10 years, the NMCP has performed creditably, reducing malaria-related mortality and morbidity. Yet in 2013, for instance, malaria was responsible for more than 11 million reported outpatient health facility visits and 2,506 deaths. However, only 48% of the 11 million reported cases were tested either by microscopy or rapid diagnostic tests and, of those tested, 1,639,451 cases were confirmed positive (24). The NMCP's ‘test, treat, and track’ policy recommends artesunate-amodiaquine as a first-line drug and artemether-lumefantrine as an alternative for uncomplicated malaria treatment. Even though an IPT policy during pregnancy exists, using sulphadoxine-pyrimetamine, Ghana lacks an IPT policy against malaria in infants and schoolchildren (1). The lymphatic filariasis control programme of the GHS has been distributing ivermectin and albendazole annually in all primary schools in the Upper East Region of Ghana, but praziquantel and mebendazole are added occasionally for the control of schistosomiasis and STHs in the KNDs (33).

Ghana has a fee-free primary school education policy. Ghana's educational system has expanded, and more than 90% of eligible children are enrolled in primary schools. Yet, high teacher absenteeism, low teacher-pupil contact time, poor physical infrastructure, and lack of teaching and learning aids, remain challenges and negatively impact educational outcomes (34). The population for this study consisted of class three schoolchildren in the Naaga, Biu, Akurugu Daabo, Kayoro, Katiu, and Nania communities of the KNDs in Ghana.

### Study design and interventions

The study was a three-arm, open-label, before-after intervention study including a control arm. The study design, as agreed on with the GHS managers at the regional and district health directorates was to be as close as possible to ‘real-life’ implementation of the mass drug administration of ivermectin, albendazole, and praziquantel, and was to explore the feasibility of combining IPT of malaria with schistosomiasis and/or STH chemotherapy in school health programmes and assessing the health and cognitive impacts. The four parts of the study were 1) a baseline study to assess the situation in the study area prior to interventions; 2) an evaluation of the impact of the intervention on health and educational capabilities of the schoolchildren; 3) a safety and tolerability study recording adverse drug reactions (ADRs), adverse events (AEs), and serious adverse events (SAEs); and 4) an end-term study to evaluate and document the findings from the various sub-studies. The study sought to answer the following research questions:

1. Will IPT with artemether-lumefantrine add to the effect of albendazole and praziquantel in the reduction of the prevalence of the anaemia, malaria, and schistosomiasis burden 1 year after mass treatment?
2. Will IPT with artemether-lumefantrine combined with deworming improve sustained attention and recall better than anthelminthic given alone?
3. Is IPT with artemether-lumefantrine as effective as praziquantel in reducing the prevalence and intensity of schistosomiasis?
4. Is artemether-lumefantrine combined with albendazole and praziquantel safe and tolerated among schoolchildren?

The three arms of the study were:

Arm 1: Artemether-lumefantrine and albendazole (131 children in Naaga and Kayoro primary schools)

Arm 2: Artemether-lumefantrine, albendazole, and praziquantel (90 children in Biu and Katiu primary schools)

Arm 3: Praziquantel and albendazole (127 children in Akurugu Daabo and Nania primary schools)

Study Arms 1 and 2 were the intervention arms, while Control Arm 3 was the control arm.

### Inclusion and exclusion

Only primary schoolchildren in class three with no known history of allergy or adverse reactions to any of the study medications were included in this study. We included only class three based on the WHO recommendation for community-based evaluations of schistosomiasis and STH control programs (7). We excluded class three schoolchildren who were grossly sick (including children with severe malaria and those severely anaemic) or those whose parents did not grant informed consent.

### Selection of participating schools

The Navrongo Demographic Surveillance System, run by the Navrongo Health Research Centre (NHRC), serves the KNDs and demarcates the districts into five geographic zones (east, west, north, south, and central) relative to the location of the communities to Navrongo, the traditional capital city of the KNDs. We used a purposive sampling technique. We selected the six primary schools because they were at least 10 km from Navrongo, and they had a community clinic or community health and planning services (CHPS) compound. Conveniently, two primary schools in the south (Naaga and Biu), two in the west (Kayoro and Katiu), and one school each from the east (Akurugu Daabo) and north (Nania) were selected for the survey. No primary schools were selected from the central zone due to repeated administration of praziquantel and mebendazole by the District Health Directorate in Navrongo. This ensured a fair representation of the districts based on previous data on *Schistosoma haematobium* and malaria transmission (30–33). Using a lottery, we randomly selected Kayoro and Naaga for Study Arm 1, Katiu and Biu for Study Arm 2, and Nania and Akurugu Daabo for Control Arm 3.

### Determination of malaria parasitaemia, haemoglobin levels, and anaemia

Thick blood films from finger prick blood were prepared, air-dried, Giemsa-stained, and examined for malaria parasites against 200 white blood cells (WBCs) by two experienced microscopists at the regional hospital laboratory in Bolgatanga. One hundred high-power fields were counted before a film was declared negative. A third experienced microscopist in the NHRC conducted a quality assurance reading of 10% of the slides. In the case of a discrepancy, slides were recounted until a consensus was obtained. Haemoglobin (Hb) was measured with a HemoCue^®^ photometer, and anaemia was defined as an Hb concentration <11.0 g/dl.

### Sustained attention testing

To determine sustained attention, the code transmission test (CTT) battery of the Test of Everyday Attention for Children was adapted (35). A list of digits were prerecorded on a tape and read one digit per second. Grouping children in 10 per session, the tape was played and children listened out for a ‘code’, which was the consecutive occurrence of the digit 5, and wrote down the number preceding the code. Two demonstration sessions were undertaken prior to the test. CTT ‘A’ was administered before interventions in June 2011, and CTT test ‘B’ at the end of the study in June 2012. Scores were based on the number of correct codes identified (one to five). To complement CTT, we adapted and used the mini-mental state tests in which study participants spelt ‘WORLD’ backwards (36, 37). We asked each participant, for example, to spell the word ‘chalk’ backwards. The score was the number of letters in the correct order spanning zero to five (e.g. klahc=5, klkac=3, etc.).

### Recall testing

We determined recall by asking study participants to recall the names of three objects (lizard, pencil, and mango) previously mentioned by the investigator after 10 min. A score of one was allotted to each correct answer up to a maximum of three (37, 38).

### Drug administration

We administered artemether-lumefantrine (20/120 mg), in a standard 3-day schedule according to body weight, twice per day in June, September, and December 2011. Albendazole (400 mg) was given in June and December 2011, based on the District Health Directorates’ STHs control plans. Praziquantel, 40 mg/kg body, was given only in June 2011. Each study participant ate a meal about 30 min prior to interventions, and all medicines were administered with a cup of clean water. All children were observed for about an hour after drug intake. We used both active and passive follow-up to collect information on ADRs, SAEs, and AEs from each study child from the day medicines were administered up to 28 completed days after treatment. Active follow-up involved community health officers and field workers visiting each study participant daily at home to collect information on ill health after interventions. Passive follow-up involved visiting community clinics and/or making telephone calls to parents and gathering information on ill-health events after interventions.

### Ethical considerations

Ethical clearance was obtained from the Navrongo Health Research Centre Institutional Review Board (NHRC-IRB), Navrongo, Ghana, in January 2011, reference NHRCIRB098. The study was registered with ClinicalTrials.gov (Identifier: NCT01459146). The study was conducted in accordance with the Helsinki Declaration of 1975, as revised in 2008.

Prior to data collection, consultations with programme heads for both NMCP and NTDs were held to present and discuss the study design and implementation strategy according to existing guidelines (38–40). Permission was sought from regional and district health and education directors and managers as well as community chiefs and leaders. Community meetings and durbars were held to discuss the study with community members in all six participating communities; clarifications were made and answers to questions raised were given. The NHRC-IRB approved written informed consent and assent forms that were in English and in the three local languages: Kasem, Nankam, and Buli. A community health officer and an NRHC ‘field worker’ (both trained in the IRB-approved study protocol) visited parents/guardians in their homes, explained the study to them in the local languages, and invited them to visit the community clinic or CHPS compound the next day. In the clinic/CHPS, the study physician presented the informed consent form to each parent/guardian who, upon agreement, either signed or thump-printed the form. Parents/guardians and children were told that participation in the study was voluntary and that they could withdraw from the study at any time.

### Data management

Data were entered into Excel files independently by two data entry officers of the NHRC, then merged and harmonized, cleaned, and analysed with STATA^®^ version 11 SE (Stata Corp., College Station, TX, USA). Means and standard deviations were constructed for continuous variables such as Hb level, CTT, and recall test scores. With categorical variables such as sex, presence of malaria, and anaemia, we computed proportions and 95% confidence intervals. Chi-square and Fisher's test were used to compare proportions. All statistical tests were two-sided at a significance of alpha <0.05.

## Results

### Characteristics of study participants

Overall, 378 class three schoolchildren were registered, but 10 parents (of six boys and four girls; three in Biu, two each in Kayoro and Naaga, and one each in Katiu, Nania and Akurugu Daboo primary schools) declined consent due to the fear of side effects. Eight children who were grossly ill (five with severe anaemia and three with severe malaria) were excluded from the interventions and were referred to the War Memorial Hospital for treatment. A total of 360 parents/guardians gave informed consent, and all children assented to participate. However, 12 children (three in Study Arm 1, four in Study Arm 2, and five in Control Arm 3) dropped out because their parents had relocated to communities outside the KNDs.

The characteristics of the study participants are summarized in Table 1.

**Table 1:** Characteristics of class three schoolchildren enrolled by study arm in KNDs, Ghana, 2011

Characteristics	Arm 1 (*N*=131)	Arm 2 (*N*=90)	Arm 3 (*N*=127)
Sex			
Boys (*n*=167)	73 (40.6)	38 (21.1)	56 (31.1)
Girls (*n*=181)	58 (34.5)	52 (70.3)	71 (42.3)
Mean age in years, (%)			
6–9	28 (21.4)	26 (28.9)	37 (29.1)
10–12	64 (48.9)	49 (54.4)	70 (55.1)
13–15	39 (29.8)	15 (16.7)	20 (15.7)
Weight (kg)			
Mean/SD	29.8/5.9	31.8/4.4	29.3/5.5
Range	20.8–43.0	25.7–46.4	17.6–50.9
Height (cm)			
Mean/SD	142.6/10.9	141.1/6.3	139.6/9.5
Range	122–168	126–155	120–165
Lives with at least one parent: yes	128 (97.7)	87 (96.7)	124 (97.6)
Mother's educational attainment			
None	22 (16.8)	16 (17.8)	26 (20.5)
Primary	47 (35.9)	30 (33.3)	48 (37.8)
Secondary	34 (25.9)	25 (27.8)	48 (37.8)
Tertiary	19 (14.5)	12 (13.3)	21 (16.5)
Number of children who slept under a bed net the previous night (%)	41 (31.3)	33 (36.7)	52 (40.9)
Number of children who took an antimalarial within 2 weeks (%)	13 (9.9)	9 (10.0)	18 (14.2)
Number of children who reported seeing bloody urine (%)	35 (26.7)	8 (8.9)	23 (18.1)
Number of children who reported seeing bloody stools (%)	9 (6.9)	5 (5.6)	12 (9.4)
Number of children who swam in a pond or stagnant body of water (%)	33 (25.2)	20 (22.2)	52 (40.9)

SD=standard deviation.

### Impact of interventions on malaria parasitaemia

Table 2 shows the prevalence of malaria parasitaemia and moderate anaemia at baseline. After intervention, significant changes in malaria parasite prevalence were observed in Study Arms 1 and 2 but not in the Control Arm 3, as shown in Table 3.

**Table 2:** Distribution of malaria parasitaemia and moderate anaemia in 348 class three schoolchildren in KNDs, Ghana, in June 2011

Primary school (number of class three children examined)	Number with malaria parasitaemia, percentage (95% CI)	Number moderately anaemic, percentage (95% CI)
Naaga (61)	13, 21.3 (13.7–35.5)	21, 34.4 (22.4–46.5)
Kayoro (70)	15, 21.4 (14.1–34.4)	10, 14.3 (6.0–22.6)
Katiu (40)	2, 5.0 (0.8–15.8)	10, 25.0 (11.4–38.6)
Biu (50)	18, 36.0 (28.1–55.9)	14, 28.0 (15.4–40.6)
Akurugu Daboo (74)	21, 28.4 (19.3–40.3)	16, 21.6 (12.1–31.1)
Nania (53)	21, 39.6 (28.1–54.9)	19, 35.8 (22.8–48.9)

**Table 3:** Effects of interventions on malaria parasitaemia and anaemia in class three schoolchildren in KNDs, Ghana, by study arm

Variable	Arm 1 (*N*=131)	Arm 2 (*N*=90)	Arm 3 (*N*=127)
Prevalence of malaria parasitaemia			
Before interventions	24.2, (2.5)	26.7, (4.1)	34.7, (3.4)
After interventions	9.0, (1,4)	10.9, (2.5)	31.6, (1.7)
*p*	0.001	0.001	0.148
Malaria parasite density (geometric mean) per 800 WBCs, (SD)			
Before intervention	346, (38)	121, (76)	535, (136)
After intervention	212, (24)	114, (32)	368, (21)
*p*	0.531	0.741	0.350
Mean Hb (g/dl), (SD)			
Before interventions	11.7, (2.5)	11.7, (1.2)	11.6, (1.1)
After interventions	11.8, (1.6)	11.6, (1.1)	11.6, (1.2)
*p*	0.745	0.631	0.701
Prevalence of anaemia, (95% CI)			
Before interventions	19.8, (16.0–29.2)	26.7, (13.3–31.0)	28.3, (6.1–43.2)
After interventions	12.2, (8.3–14.7)	21.1, (15.4–28.5)	18.1, (15.8–27.6)
*p*	0.073	0.151	0.049

WBCs=white blood cells.

### Impact of interventions on anaemia prevalence

There were no significant changes in Hb levels within the study arms (Table 3). However, anaemia prevalence reduced from 19.8% (26/131) at baseline in Study Arm 1 to 12.2% (16/131) after intervention (reduction: 38.4%). In Study Arm 2, anaemia prevalence reduced from 26.7% (24/90) before interventions to 21.1% (19/90) after interventions (reduction: 20.7%). In Control Arm 3, anaemia prevalence reduced from 28.3% (36/127) before treatment to 18.1% (23/127) after treatment (reduction: 36.0%). Comparing anaemia prevalence risk reduction in Study Arms 1 and 2 to that of Control Arm 3, the relative risks were 1.07 and 0.58, respectively, but anaemia prevalence risk reduction between Study Arms 1 and 2 was 1.38.

### Impact of interventions on sustained attention

The impact of interventions on sustained attention scores, represented by the mean CTT scores, is presented in Table 4. Scores improved by 10.4% after interventions in Study Arm 1 and 20.5% in Study Arm 2. In Control Arm 3, however, mean CTT scores only improved by 5.75% post-intervention. When we compared the mean CTT score improvements in Study Arms 1 and 2 to that of Control Arm 3, the rate ratios are 1.81 and 3.57, respectively.

**Table 4:** Impact of interventions on code transmission test (CTT) scores in class three schoolchildren in KNDs, Ghana, 2011 and 2012

Study arm	Variable	Mean CTT score before interventions in June 2011 (95% CI)	Mean CTT score after interventions in June 2012 (95% CI)	*p*
Arm 1 (AL+ALB)	Boys	3.14 (2.95–3.33)	3.47 (3.26–3.69)	0.02
	Girls	3.23 (3.10–3.36)	3.63 (3.46–3.80)	0.01
	Total	3.18 (2.94–3.38)	3.55 (3.20–3.91)	0.01
	6–9 years	3.24 (3.06–3.42)	3.58 (3.26–3.91)	0.06
	10–12 years	3.23 (3.08–3.37)	3.58 (3.38–3.77)	0.01
	13–15 years	3.14 (2.89–3.38)	3.54 (3.29–3.78)	0.01
Arm 2 (AL+ALB+PZQ)	Boys	2.83 (2.63–3.02)	3.49 (3.28–3.69)	0.01
	Girls	2.84 (2.58–3.10)	3.64 (3.44–3.84)	0.01
	Total	2.83 (2.52–3.14)	3.56 (3.25–3.86)	0.01
	6–9 years	2.66 (2.31–2.99)	3.44 (3.19–3.69)	0.01
	10–12 years	2.87 (2.68–3.06)	3.60 (3.41–3.79)	0.01
	13–15 years	3.07 (2.74–3.39)	3.56 (2.99–4.11)	0.11
Arm 3 (AL+PZQ)	Boys	3.08 (2.92–3.25)	3.14 (2.92–3.37)	0.67
	Girls	2.82 (2.61–3.03)	3.13 (2.81–3.44)	0.11
	Total	2.95 (2.58–3.29)	3.13 (2.75–3.48)	0.09
	6–9 years	3.05 (2.83–3.28)	3.15 (2.79–3.46)	0.72
	10–12 years	2.91 (2.73–3.08)	3.18 (2.94–3.42)	0.07
	13–15 years	3.07 (2.62–3.51)	2.83 (2.04–3.62)	0.54

AL=Artemether-lumefantrine; ALB=albendazole; PZQ=praziquantel; CI=confidence interval.

### Impact of interventions on recall test scores

Table 5 presents the impacts of interventions on mean recall test scores among schoolchildren. Mean recall test scores improved by 16.9% in Study Arm 1; but in Study Arm 2, recall test scores improved by 27.9% compared to 18.3% improvement in Control Arm 3. Comparing the improvement in mean recall test score for Study Arms 1 and 2 to that of Control Arm 3, the improvement rate ratios were 1.08 and 1.52, respectively.

**Table 5:** Impact of interventions on mean recall test scores in class three schoolchildren in KNDs, Ghana, 2011 and 2012

Study arm	Variable	Mean recall test score before interventions in June 2011 (95% CI)	Mean recall test score after interventions in June 2012 (95% CI)	*p*
Arm 1 (AL+ALB)	Boys	2.05 (1.89–2.21)	2.71 (2.54–2.87)	0.01
	Girls	2.09 (1.98–2.21)	2.08 (2.66–2.91)	0.01
	Total	2.07 (1.82–2.34)	2.49 (2.32–2.96)	0.01
	6–9 years	2.28 (2.03–2.53)	2.67 (2.35–2.97)	0.05
	10–12 years	1.98 (1.85–2.12)	2.78 (2.64–2.91)	0.01
	13–15 years	2.09 (1.95–2.24)	2.75 (2.58–2.92)	0.01
Arm 2 (AL+ALB+PZQ)	Boys	1.89 (1.73–2.04)	2.54 (2.35–2.73)	0.01
	Girls	1.92 (1.69–2.14)	2.76 (2.58–2.94)	0.01
	Total	1.91 (1.63–2.16)	2.65 (2.31–2.97)	0.01
	6–9 years	2.14 (1.94–2.33)	2.67 (2.43–2.91)	0.01
	10–12 years	1.80 (1.62–1.98)	2.54 (2.35–2.74)	0.01
	13–15 years	1.73 (1.40–2.06)	2.89 (2.63–3.15)	0.01
Arm 3 (AL+PZQ)	Boys	1.98 (1.83–2.14)	2.38 (2.19–2.56)	0.01
	Girls	1.86 (1.71–2.01)	2.31 (2.09–2.53)	0.01
	Total	1.92 (1.68–2.18)	2.35 (2.04–2.61)	0.01
	6–9 years	2.05 (1.83–2.28)	2.38 (2.10–2.65)	0.07
	10–12 years	1.83 (1.69–1.96)	2.39 (2.22–2.55)	0.01
	13–15 years	2.13 (1.78–2.45)	2.00 (1.34–2.66)	0.67

AL=Artemether-lumefantrine; ALB=albendazole; PZQ=praziquantel; CI=confidence interval.

## Discussion

Study findings showed that co-administration of IPT for malaria and deworming was associated with a significant reduction in malaria parasite prevalence after interventions in Study Arms 1 and 2 when compared to Control Arm 3, where no antimalarial drug was given. This result is consistent with findings from previous IPT trials involving schoolchildren elsewhere in sub-Saharan Africa in which IPT reduced malaria prevalence remarkably (10, 11, 13–15).

However, the impact of IPT on anaemia varied: some studies observed significant reductions in anaemia prevalence, although others did not (10, 11, 13–15). In our opinion, artemether-lumefantrine could be the medicine exerting this significant antimalarial effect because it is already known that anthelminthic drugs, such as albendazole and praziquantel, have no impact on malaria parasites (41). However, the slight reduction in malaria parasite prevalence observed in Control Arm 3 could be due to the use of insecticide-treated bed nets, self-medication with antimalarial medicines, treatment with antimalarial medicines by community-based agents and chemical sellers, or to the year-to-year variation in transmission level of the study area (29–32, 34). These same factors could be responsible for the variations in malaria parasite prevalence observed between Study Arms 1 and 2 after the interventions.

The study findings showed that the co-administration of IPT and deworming was associated with no significant reductions of anaemia prevalence in schoolchildren in the study area. This finding concurs with the results of previous studies in Ghana, Kenya, and Uganda in which IPT and deworming did not significantly reduce anaemia prevalence – even though some reductions in anaemia prevalence were observed after treatment (10, 12, 14, 15, 42). However, anaemia prevalence reduction was slightly higher in the control arm and in Study Arm 1 than in Study Arm 2. This could have been due to many more parasites (for instance, hookworms and *S. mansoni*) that were responsible for anaemia in the control group and in Study Arm 1 and that were more sensitive to the interventions in Study Arms 1 and 3 than in Study Arm 2.

Although our results could be used to support advocacy efforts for school-age children in regions of the world where malaria, schistosomiasis, and STH coexist who might benefit from both IPT and deworming interventions, we are also of the opinion that other factors that might not have responded to the deworming and IPT interventions might contribute to anaemia in the study area. Poverty, malnutrition, and genetic predispositions to sickle cell disease and glucose-6-phosphatase dehydrogenase (G6PD) deficiencies, for example, are common in the study area (25, 26, 34, 43).

The results of our study suggests that co-administered IPT and deworming could significantly improve schoolchildren's sustained attention and recall. Although these findings concurred with previous studies in which IPT was associated with improved sustained attention and recall (10, 44), our results suggest that schoolchildren could derive more benefits involving sustained attention and recall when artemether-lumefantrine, praziquantel, and albendazole are co-administered. With respect to the differences in sustained attention and recall test scores between Study Arms 1 and 2, even though we are not certain what was responsible for these differences, we think that the addition of praziquantel could be the reason because it afforded children treatment for schistosomiasis in Study Arm 2, but this was not the case in Study Arm 1 (42).

### Strengths and limitations

The high prevalence of parasitic infections (including malaria, schistosomiasis, and STHs) in school-age children and the associated effects on their health (including anaemia and growth stunting) and education is a matter of great concern (7–9, 25, 31). Even though an integrated control approach has been advocated for malaria and NTDs, this study (to the best of our knowledge) was the first attempt in sub-Saharan Africa at integrated control of malaria, schistosomiasis, and STHs using co-administered medicines.

Because of limitations due to funding constraints and expansion to accommodate other thematic areas, our study, which was initially designed as a randomized control trial (RCT), had to be changed to an open-label before-after intervention study with a control arm. One key consideration that influenced our dropping the RCT method was that disease control activities in the study area were planned activities not random events. Other considerations were: 1) the purpose of most RCTs is to determine an intervention's net benefit under ideal circumstances (efficacy) either to satisfy food and drug authorities’ marketing approval requirements or to provide insights into a disease's aetiology and underlying mechanisms; 2) RCTs are conducted under tightly controlled clinical conditions that are consequently less likely to reflect the conditions under which interventions are used in common clinical practice for parasitic disease control; 3) the results from RCTs frequently do not reach their potential value for health care decision making, which is a serious waste of resources (45–48). On the other hand, the choice of a before-after study with a control arm was informed by our desire to produce results that would be clinically relevant and timely for policy decisions on integrated parasitic disease control in the study area. Although we concede that our choice was not the best for optimal evidence generation, before-after studies with control groups may be especially useful for rapidly evolving interventions and comparative effectiveness research trials designed for dynamic, unique needs of community medical practices and may be consistent with the concept of a ‘learning healthcare system’ in that it allowed flexible, adaptive, cumulative learning to be incorporated while the trial is being conducted. It is also useful for studying patient or sub-group characteristics that predict response to alternative management strategies (45–48).

Another weakness of the study as the purposive sampling of primary schools and the choice of class three schoolchildren, which may have affected the generalizability of the results. Purposive sampling was determined by the fact that NTD control programmes in Ghana are almost always targeted at specific eco-epidemiologic settings where a disease is most common and hardly are selected by chance! We, however, chose class three schoolchildren because WHO recommends using this age group for intervention evaluation for the control of STHs and schistosomiasis (3, 4, 7).

Although our findings suggested that treating malaria, schistosomiasis, and STHs could be associated with improved sustained attention and recall, poor performance by children in tests of sustained attention and recall must not necessarily be interpreted as frontal-parietal brain or basal brain impairments. It should be noted that, as children grow, their ability to adapt to and focus on classroom learning and to withstand a test of sustained attention improves; they learn new things and gain new experiences that promote their vigilance and recall. Therefore, children taking a similar test one year later are expected to perform better (33–35). Despite the aforementioned limitations, our findings are important and could contribute vital information supporting efforts toward integrated control of malaria, schistosomiasis, and STHs in schoolchildren.

## Conclusions

The results of this study suggest that, in an area where malaria, schistosomiasis, and STHs coexist, co-administration of IPT of malaria and deworming to schoolchildren may significantly reduce malaria prevalence, contribute to a reduction in anaemia prevalence, and significantly improve sustained attention and recall. The best results may be obtained when artemether-lumefantrine, albendazole, and praziquantel are co-administered. Further research is needed to confirm these findings, to assess the effects of the interventions on schistosomiasis and STHs, and to study the safety and tolerability of these co-administered medicines.

## Paper context

Even though parasitic diseases such as malaria, STHs, and schistosomiasis commonly infect school-age children in parts of Ghana and sub-Saharan Africa, little is known about the benefits of integrated control of these parasitic diseases on anaemia, sustained attention, and recall in schoolchildren. This paper seeks to share information on some of the health and educational benefits derived from the implementation of integrated control of malaria and helminth infections in Northern Ghanaian schoolchildren to guide public health practitioners, managers, and policy-makers. This study was designed to answer the research question: ‘To what extent would co-administered IPT with artemether-lumefantrine and albendazole plus or minus praziquantel reduce anaemia prevalence and improve sustained attention and recall in schoolchildren compared with albendazole plus praziquantel as control?’
