# Corrigendum: Impact of combined intermittent preventive treatment of malaria and helminths on anaemia, sustained attention, and recall in Northern Ghanaian schoolchildren

**DOI:** 10.3402/gha.v9.33548

**Published:** 2016-10-21

Regarding the paper titled: ‘Impact of combined intermittent preventive treatment of malaria and helminths on anaemia, sustained attention, and recall in Northern Ghanaian schoolchildren’ by Ernest Cudjoe Opoku, Annette Olsen, Edmund Browne, Abraham Hodgson, John K. Awoonor-Williams, Lawrence Yelifari, John Williams and Pascal Magnussen

Published in *Global Health Action* (section “Original Articles”) 14 Sep 2016.

Citation: Glob Health Action 2016, 9:32197 - http://dx.doi.org/10.3402/gha.v9.32197

The beginning of the Results section in the Abstract currently reads:

We observed significant malaria parasite prevalence reductions of 62.8 and 59.2% in Study Arm 1 from 24.2 to 9.0%, *p*<0.01, and 59.2% in Study Arm 2 from 26.7 to 10.9%, *p*<0.01), respectively, compared to 8.93% in Control Arm 3 (from 34.7 to 31.6%, *p*>0.05). Meanwhile, anaemia prevalence reduced significantly (*p*<0.01) in all three study arms after interventions by 38.4% (from 19.8 to 12.2%), 20.7% (from 26.6 to 21.1%), and 36.0% (from 28.3 to 18.1%) in Study Arms 1, 2, and 3, respectively. Although the interventions had no significant effects on Hb levels, anaemia prevalence reduced insignificantly by 38.4 and 20.7% in Study Arms 1 and 2, respectively, compared to 36.0% in Control Arm 3.

It should read:

We observed significant malaria parasite prevalence reductions of 62.8% in Study Arm 1 (from 24.2 to 9.0%, *p*<0.01), and 59.2% in Study Arm 2 (from 26.7 to 10.9%, *p*<0.01), respectively, compared to 8.93% in Control Arm 3 (from 34.7 to 31.6%, *p*>0.05). Although the interventions had no significant effects on Hb levels, anaemia prevalence reduced by 38.4 and 20.7% in Study Arms 1 and 2, respectively, compared to 36.0% in Control Arm 3.

